# Systemic Lupus Erythematosus and Myasthenia Gravis in a Male Patient: an HLA Case Investigated

**DOI:** 10.31138/mjr.32.3.285

**Published:** 2021-06-27

**Authors:** Eleni Klimi, Evangelia Kataxaki

**Affiliations:** 1 Department of Dermatology, Thriassio General Hospital, Athens, Greece; 2 Department of Rheumatology, Thriassio General Hospital, Athens, Greece

**Keywords:** Systemic lupus erythematosus, myasthenia gravis, HLA

Dear editor,

Systemic Lupus Erythematosus (SLE) and myasthenia gravis (MG) are both immune disorders mediated by the production of autoantibodies. Systemic lupus erythematosus is characterised by the development of dysregulated autoreactive B-cell derived autoantibodies directed against nuclear and cellular components, and the activation of complex inflammatory cascade, thereby resulting in multisystem organ damage.

Myasthenia gravis is an organ-specific autoimmune disease characterised by dysfunction of the neuromuscular junction, mediated by autoantibodies against the nicotinic acetylcholine receptor, the muscle-specific tyrosine kinase, or the low-density lipoprotein receptor related protein 4, resulting in muscle weakness.

Systemic lupus erythematosus can precede or follow the development of MG.^1^

Coexistence of SLE and MG is a rare phenomenon. A 55-year-old male Caucasian patient who was diagnosed 17 years ago with MG, for which thymectomy was performed, controlled with pyridostigmine 60mg, five times daily total daily dose 300mgr and azathioprine 50mgr twice daily, developed an ischemic cerebrovascular accident, arthritis, and lesions on his hands typical of discoid lupus. Antinuclear antibodies(titer1:160), SM, RNP were positive. The score, according to the recent criteria of the American Association of Rheumatologists, is 16 in total, above the 10-joint involvement score (6), discoid skin lesions score (4) and serum antibodies anti Smith score (6). An HLA typing was performed for the HLA-class I antigens with the sequence specific oligonucleotide-polymerase chain reaction (PCR) and for the HLA- class II the sequence specific primer PCR-based assay. The HLA profile for the HLA class I was A1, A2, B8, Cw7, and for the HLA class II DRB1^*^ 14, DRB1^*^ 16:02.

Systemic lupus erythematosus is an autoimmune disease with an important genetic component, as is MG.^2–3^ Myasthenia gravis mostly affects female patients, the patient of this study is a male one, and SLE usually follows thymectomy for MG because thymectomy by breaking immune tolerance promotes production of autoantibodies, and, consequently, development of SLE. Evidence exists to support the genetic predisposition of HLA –DRB1 gene polymorphisms to SLE.^2–3^ DRB116:02 which was found in our patient is associated with autoimmune disorders with production of autoantibodies including both SLE and MG.^4^ Positivity was also detected for the DRB114 allele, which was recently identified as a risk factor for the late onset MG in Caucasians.^5^ A2 and Cw7 alleles have not so far been correlated with any autoimmune disorder. The A1B8 haplotype found in the patient was previously detected in a French study, in three out of seven patients presenting both MG and SLE.^6^

A limitation of this study is that HLA investigation was conducted in one patient; however, it confirms the results of other studies, DRB114positivity for MG, DRB116:02 for SLE and MG. The most interesting finding is the association A1B8, which has been previously described only once. Further larger studies are needed to confirm if this association may be a biomarker for both MG and SLE.

**Figure 1.**
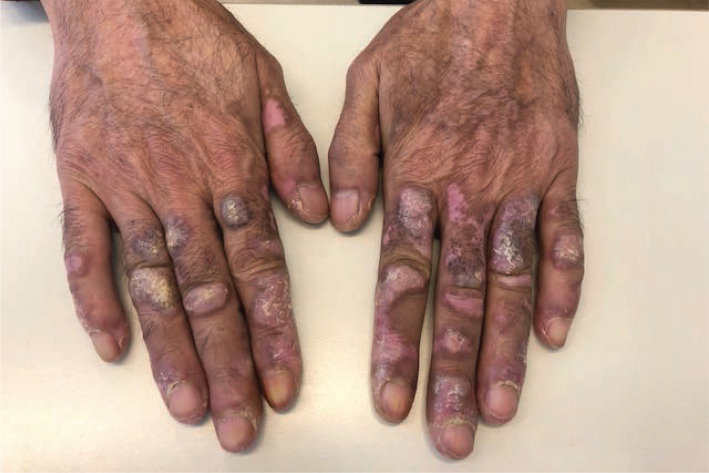
Discoid lupus erythematosus on hands.
